# Dissipative Coupling of Fluid and Immersed Objects for Modelling of Cells in Flow

**DOI:** 10.1155/2018/7842857

**Published:** 2018-09-27

**Authors:** Martin Bušík, Martin Slavík, Ivan Cimrák

**Affiliations:** University of Žilina, Univerzitná 8215/1, Žilina 01026, Slovakia

## Abstract

Modelling of cell flow for biomedical applications relies in many cases on the correct description of fluid-structure interaction between the cell membrane and the surrounding fluid. We analyse the coupling of the lattice-Boltzmann method for the fluid and the spring network model for the cells. We investigate the bare friction parameter of fluid-structure interaction that is mediated via dissipative coupling. Such coupling mimics the no-slip boundary condition at the interface between the fluid and object. It is an alternative method to the immersed boundary method. Here, the fluid-structure coupling is provided by forces penalising local differences between velocities of the object's boundaries and the surrounding fluid. The method includes a phenomenological friction coefficient that determines the strength of the coupling. This work aims at determination of proper values of such friction coefficient. We derive an explicit formula for computation of this coefficient depending on the mesh density assuming a reference friction is known. We validate this formula on spherical and ellipsoidal objects. We also provide sensitivity analysis of the formula on all parameters entering the model. We conclude that such formula may be used also for objects with irregular shapes provided that the triangular mesh covering the object's surface is in some sense uniform. Our findings are justified by two computational experiments where we simulate motion of a red blood cell in a capillary and in a shear flow. Both experiments confirm our results presented in this work.

## 1. Introduction

Deformation of elastic membranes due to an external shear flow or interactions with other such objects is an important problem in basic research as well as in biomedical applications. One of the most pronounced examples is the red blood cell (RBC) with its membrane composed of a lipid bilayer and a cytoskeleton. This membrane behaves as a viscoelastic material with property of area-conservation as described by Skalak in [1]. Elastic properties of RBC have significant effect on the physiological cell functions, and it also influences the rheology of the whole blood [2]. Moreover, the elasticity plays crucial role in the flow of RBCs inside microfluidic devices as demonstrated by Fedosov in [3]. Understanding of the dynamics of RBC is thus of great interest. However, experiment-based studies of RBC mechanics are usually difficult to perform due to the small RBC dimensions. Here, computational modelling serves as a good alternative.

Most membrane models are either derived from continuum laws or based on spring networks. Here, we focus on the latter due to their simplicity and similarity to the cytoskeleton. The membrane itself is usually described by a triangular mesh of interconnected points with elastic forces between them defining the elasticity [4].

The flow of the fluid in which the cells are immersed is usually computed from governing equations for fluid dynamics, for example, the Navier–Stokes equations for incompressible flow [5]. Recently, a lot of attention got the lattice-Boltzmann method for its relatively simple implementation while preserving high accuracy for low and moderate Reynolds numbers as shown by Chen in [6]. In this method, the boundaries are often implemented by the bounce-back rule [7] which can be extended for moving boundaries of solid objects. For deformable objects, the combination of mesh-based methods for the object description and the lattice-Boltzmann method for fluid computation has been recently used to model cell flow. Some examples of such models include work of Kruger, Aidun, and other authors [8, 9, 10, 11, 12, 13, 14] and references therein.

The crucial element in such models of the cell flow is the coupling of the membrane mesh and the underlying velocity field of the fluid. An efficient way of imposing these boundary conditions is via immersed boundary method (IBM), first introduced by Peskin [15]; for the overview of different types of IBM, we refer to work of Mittal and Iaccarino [16]. Here, the no-slip boundary condition is imposed on the membrane of the cell, and the velocity of the mesh point **X** is set to be the velocity of the surrounding fluid **u**. Because of the fixed fluid discretisation, the fluid velocity at the position **X** is obtained from convolution with suitably chosen *δ* function

(1) $$\begin{array}{c} \frac{\partial X}{\partial t}\left(t\right)=u\left(X\left(t\right)\right)=\int u\left(x\right)\delta \left(x-X\left(t\right)\right)\text{ d}x. \end{array}$$

The concrete form of the delta function influences numerical properties of the method. Yang et al. [17] use a smoothing technique for discrete delta function to avoid nonphysical oscillations of the volume force, appearing in the governing equations. In general, the right choice of the proper *δ* function is a challenging task.

The IBM does not account for the mass of the boundary. The mesh points are massless and in situations when mass of the membrane does play a role, the use of the IBM is limited. A variant of IBM has been introduced by Kim and Peskin [18], where the authors account for the mass of the membrane by introducing a dual mesh which carries the mass. The points of the dual mesh move according to the Newton equations of motion and are linked to the original mesh by stiff springs.

### 1.1. Dissipative Coupling

In this article, we elaborate a different approach of the coupling between the membrane mesh and the fluid using dissipative force coupling. This so-called force-coupling algorithm was first introduced by Ahlrichs and Dunwegin [19] and later adapted by Lobaskin and Dunwegin [20] to model colloidal particles. This model has been named the raspberry model and was recently improved by de Graaf and co-workers [21, 22]. In these studies, the hydrodynamic properties of colloidal particles have been thoroughly studied. Besides spherical objects, nonspherical objects were also considered in the latter studies.

Since its inception, the force-coupling algorithm has seen several improvements in terms of accuracy and flexibility. Ladd et al. [23] have devised a proper discrete integration scheme for the coupled system. A second-order accurate discretisation and a unified formalism for fluid-particle interactions including dissipative coupling, immersed boundary method, and external boundary were derived by Schiller [24].

This approach introduces a friction coefficient that needs to be properly calibrated. In the detailed analysis of force-coupling algorithm [21, 22], Fisher and coworkers examine the accuracy with which the raspberry method is able to reproduce Stokes-level hydrodynamic interactions when compared with analytic expressions for solid spheres in simple-cubic crystals. In their work, they focus on determination of the fit parameter, the effective hydrodynamic radius.

We studied this method in [25]; however, at that time, we did not properly analyse the emerging friction coefficient. We developed the complete model for modelling deformable objects such as capsules and vesicles in [25]. Its software implementation was described in [26]. The model is based on the lattice-Boltzmann method for fluid dynamics coupled with the immersed boundary method for the membrane description. The fluid-membrane coupling is provided by the dissipative force-coupling algorithm between the fixed lattice nodes and nodes of the triangular mesh for the cell membrane. The coupling force is proportional to the difference between the fluid and object's velocities. Similar idea was used by Bigot in case of rigid bodies [27]. The force is added to the governing equations, both for fluid and for the membrane, in such a way that it penalises velocity difference. This approach mimics the no-slip condition.

The coupling force is scaled by a prefactor called friction coefficient denoted as *ξ*.

Similar methods for modelling deformable objects have been proposed. Reasor et al. [13] introduce a spectrin-link red blood cell membrane method coupled with a lattice-Boltzmann fluid solver. They implement different force-coupling interactions between the fluid and structure. They interpolate the force due to the bounce-back operation between the interior and exterior nodes onto the spectrin-link triangulated surface. This operation is performed along the direction perpendicular to the membrane surface. A different approach was used by Krueger et al. [10, 11] where finite element methods give the description of object's deformation.

In the present work, we focus on proper determination of the friction coefficient. It is a purely phenomenological term, and we determine its value in such a way that the movement of objects corresponds to reality. As a ground truth, we take a physically relevant experiment, and we determine the friction coefficient for one specific triangulation of this object. This calibrated value will serve as a reference starting point for any other mesh with different mesh density or size of the object. We derive the explicit formula for computation of friction coefficient based on this reference value.

The paper is organised as follows. In Section 2, we briefly describe three main parts of our model: fluid solver based on the lattice-Boltzmann method, cell membrane model based on spring networks, and the coupling of both models. Next, in Section 3, we introduce two scenarios for experiments which can be simulated with our computational model. The outcomes of these experiments may be predicted by theoretical calculations and subsequently compared with simulation results obtained by our model.  Section 4 describes a series of simulations, the results of which are used to calibrate the friction coefficient for the reference object. In Section 5, we propose a hypothesis about the friction coefficient recalculation for an object with different shape, size, and discretisation. The hypothesised expression is verified in Section 6 for ellipsoidal objects. In Section 7, we address the effect of fluid viscosity on the value of friction coefficient. To demonstrate the capability of the model with correct fluid-structure interaction, in Section 8 we present computational study of red blood cell flowing in a tube with diameter comparable with the size of the cell. This example shows that the cell deforms to a parachute-like shape reported in experimental observations. In Section 9, we provide validation of the derived expression for the friction coefficient using real biological experiments of red blood cells immersed in a shear flow. We consider cell's rotational frequency and compare whether simulated frequency corresponds to the measured one. In concluding Section 10, we discuss the practical outcomes of acquired results for simulations, especially simulations of blood flow.

## 2. Model Description

Our computational model consists of three parts: solver for the fluid, mechanical model based on spring networks for the cells, and coupling between the fluid and the cell.

### 2.1. Fluid Governed by the Lattice-Boltzmann Method

This method describes the fluid dynamics and is based on fictive particles. These particles propagate and collide over a fixed three-dimensional discrete lattice. The unknown variable is the particle density function *n*_*i*_(**x**, *t*) defined for each lattice point **x**, discrete velocity vector **e**_*i*_, and time *t*. We use the D3Q19 version of the lattice-Boltzmann method (three dimensions with 19 discrete directions **e**_*i*_ along the edges and diagonals of the lattice). The governing LB equations are

(2) $$\begin{array}{c} \underset{\text{propagation}}{\underset{︸}{n_{i}\left(x+e_{i}\delta_{t},t+\delta_{t}\right)=n_{i}\left(x,t\right)}}+\underset{\text{collision}}{\underset{︸}{\Delta_{i}\left(n\left(x,t\right)\right)}}, \end{array}$$

where *δ*_*t*_ is the time step and Δ_*i*_ denotes the collision operator that accounts for the difference between pre- and postcollision states and satisfies the constraints of mass and momentum conservation. In the lattice-Boltzmann method, the fluid flow needs to be evaluated with a half-step correction of the local force in order to be consistent with the Navier–Stokes equation. Therefore, the external forces can be incorporated by half-step Verlet algorithm. We refer to works of Ahlrichs and Dellar [19, 28] for details on the lattice-Boltzmann method. For computations, we use implementation of this method in scientific software ESPResSo [29]. The velocity field **u** and the density of the fluid *ρ* are evaluated from

(3) $$\begin{array}{c} \rho \left(x,t\right)=\underset{i}{\sum }n_{i}\left(x,t\right),\\ \rho \left(x,t\right)u=\underset{i}{\sum }n_{i}\left(x,t\right)e_{i}. \end{array}$$

### 2.2. Triangular Mesh and Newton Equations of Motion

Cell's membrane is covered by mesh points, linked together into a triangular mesh. Elastic properties of the cell membrane are represented with different types of force-like bonds between neighbouring mesh points. To take the mechanoelastic properties of the immersed objects into account, geometrical entities in this mesh (edges, faces, angles between two faces, etc.) are used to model stretching, bending, stiffness, and other properties of the membrane. One such example is the stretching force between two neighbouring mesh points defined as

(4) $$\begin{array}{c} f_{\text{s}}=-k_{\text{s}}\kappa \left(\frac{l}{l_{0}}\right)\left(l-l_{0}\right), \end{array}$$

where *k*_s_ is the stretching stiffness coefficient, *κ* is a nonlinear function mimicking neo-Hookean behaviour of cell's membrane, *l* is the current length of the edge between those two points, and *l*_0_ is the length in a cell's relaxed state. In the model we use for computations, there are all together five elastic coefficients: *k*_*s*_ for the shear stretching, *k*_b_ for the bending rigidity, *k*_al_ for local area expansion, *k*_ag_ for preservation of total surface, and *k*_v_ for preservation of total volume of the cells or other objects. Further details on formulas for each such elastic moduli are presented in [21]. These coefficients determine the elastic behaviour of the cell, or other objects. Implementation of this method into ESPResSo code was done in our earlier studies [26].

The sum of all such elastic forces defines force *f* exerted on mesh points. This force causes motion of the mesh point according to the Newton equation of motion.

(5) $$\begin{array}{c} m\frac{d^{2}x}{dt^{2}}=f, \end{array}$$

where *m* is the mass of the mesh point. The source of *f* is either from the abovementioned elastomechanical properties of the immersed object or from the fluid-structure interaction.

### 2.3. Coupling of the Lattice-Boltzmann Method and the Immersed Boundary Method

Equations (2) and (5) describe two different model components on two different meshes: the motion of the fluid and the motion of the immersed objects. For the coupling, we use an approach of Ahlrichs and Dunweg from [19, 30] based on a dissipative force between the fluid and the mesh points. The force exerted by the fluid on one mesh point is proportional to the difference of the velocity *v* of the mesh point and the fluid velocity *u* at the same position:

(6) $$\begin{array}{c} F=\xi \left(u-v\right), \end{array}$$

where *ξ* is a friction coefficient. *F* enters (5) as a part of *f*. The coupling is mutual so the opposite force is exerted on the fluid.

Friction coefficient does not correspond to any physical quantity and is purely phenomenological. It enforces the no-slip condition, and in the limit *ξ*⟶*∞*, the no-slip condition should be preserved.

In numerical computations, however, we need to use finite value. This value is dependent on different features. In the next sections, we determine the correct value of *ξ*.

## 3. Design of Computational Experiments for Friction Calibration

To determine the friction coefficient, we need to compare our computational approach with analytical results. We decided to set as a reference the movement of a solid spheroidal object (further called sphere) in a fluid. There are theoretical computations that give us exact solutions for the velocity of such sphere. We can then compare them with computed results using our model. This way, we can inversely get the correct value for the friction coefficient.

It is of course a priori not clear whether a hollow membrane consisting of interlinked mesh points can be compared with a rigid sphere since dissipation in the inside fluid may affect the friction. The presented simulations however show a good resemblance of the reality.

The motion of solid objects immersed in the fluid is described by Newton's second law of motion:

(7) $$\begin{array}{c} F=m\frac{dv}{dt}, \end{array}$$

where *m* is the mass and *v* is the velocity of the object. We focus on the flow with low Reynolds number, and for these, the drag force of the fluid on the objects is given by Stokes law as

(8) $$\begin{array}{c} F_{\text{d}}=6\pi \nu rv_{0}, \end{array}$$

where *r* is the radius of the object, *v* is its velocity relative to the surrounding fluid, and *ν* is the dynamic viscosity of the fluid. Theory assumes a solid rigid object immersed in an unbounded fluid. Actually, our model assumes elastic objects; however, by setting the elastic coefficients high, we can model solid objects as well. We have discussed the question of domain boundaries in [31], and as we concluded, it is sufficient to have 1 : 20 ratio between the size of the object and the size of the simulation box. This ratio is even smaller than 1 : 10 used for similar analysis in [22]. With diameter 10  *μ*m, simulation box with dimensions 200  *μ*m is suitable.

Based on this theoretical knowledge, we design two different experiments.

### 3.1. Terminal Velocity Experiment

**Figure 1:**
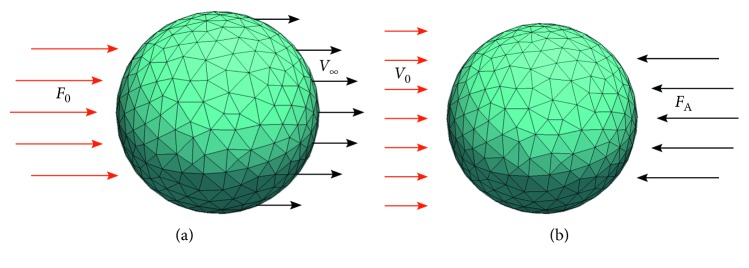
(a) Terminal velocity experiment, where force is initialised to *F*_0_ and terminal velocity *v*_*∞*_ is simulated. (b) Balancing force experiment, where velocity of fluid is initialised to *v*_0_ and adaptive force *F*_A_ is simulated.

We put a sphere into a static fluid. Constant horizontal force *F*_0_ is applied on the sphere (Figure 1(a)). The sphere accelerates, the drag force therefore increases, at some point it cancels out with *F*_0_, and sphere's velocity thus becomes stabilised at some value. We call this value *terminal velocity* and denote it by *v*_*∞*_. Terminal velocity can be derived from theoretical assumptions similarly as in [31]. We calculate terminal velocity by the following formula:

(9) $$\begin{array}{c} v_{\infty }=\underset{t⟶\infty }{\lim}v\left(t\right)=\frac{F_{0}}{6\pi \nu r}. \end{array}$$

Terminal velocity does not depend on the object's mass.

### 3.2. Balancing Force Experiment

We put a sphere into a flowing fluid with constant velocity *v*_0_. We exert a balancing horizontal force *F*_A_ in the direction opposite to the flow such that the sphere remains at its original place (Figure 1(b)). According to the theory, the balancing force exerted on the sphere in an equilibrium state equals to drag force by Stokes law. We calculate exact expression by the following formula:

(10) $$\begin{array}{c} F_{\text{A}}=6\pi \nu rv_{0}. \end{array}$$

Again, this does not depend on the object's mass.

## 4. Calibration of a Reference Sphere

At this point, we can perform computer simulations of both experiments to find a proper value of *ξ*. Two experiments should be for this purpose equivalent and serve as a double check. In the first step, we will randomly pick the value of *ξ* and we can check whether simulated values of terminal velocity in the first experiment and balancing force in the second experiment correspond to the theoretical values. If not (which is highly probable for the first shot), we adopt the value of *ξ* and try again. By this inverse process, we will be able to determine correct values of *ξ*. At the beginning, we chose the values of *ξ* with regular incrementation; then, for finer calibration, we used simple bisection/step-doubling method.

This process is quite computationally demanding. To evaluate both experiments for one single value of *ξ*, we need to perform full 3D simulation. Therefore, we first calibrate *ξ*_ref_ for a reference sphere and afterwards we derive a formula for direct computation of *ξ* for an arbitrary sphere based on the value *ξ*_ref_. For the whole calibration process, we used a simulation software ESPResSo [29], release 4.0, where the described model is implemented.

From our previous experiences with similar computations, we decided to pick the sphere with radius 4  *μ*m with 393 mesh points as a reference sphere. We use the following elastic parameters: *k*_s_=1.0, *k*_b_=0.5, *k*_al_=0.2, *k*_ag_=5.0, and *k*_v_=10.0. Such high values ensure that the sphere is not deformed and behaves like a rigid one. The mass of individual mesh points is set to 0.25 pg.

We set density of the fluid to 1025  kg · m^−3^ and dynamic viscosity to 1.5375  mPa · s, which are values of blood plasma. The spatial step of the lattice-Boltzmann grid equals 1.0  *μ*m. All simulations were performed in a cubic simulation box with edge 200  *μ*m [31]. The time step of simulations equals 0.1  *μ*s.

For terminal velocity experiment, we set the force *F*_0_=0.4  nN. From (9), we explicitly calculate the expected terminal velocity *v*_*∞*_=0.003451  m · s^−1^.

For balancing force experiment, we set the fluid velocity *v*_0_=0.010  m · s^−1^. Consequently, from (10), we compute expected balancing force *F*_A_=1.159  nN.

The boundary conditions for the fluid in terminal velocity experiment on all sides of the simulation box are set to zero. For fluid in the balancing force experiment, these conditions are set to *v*_0_.

**Figure 2:**
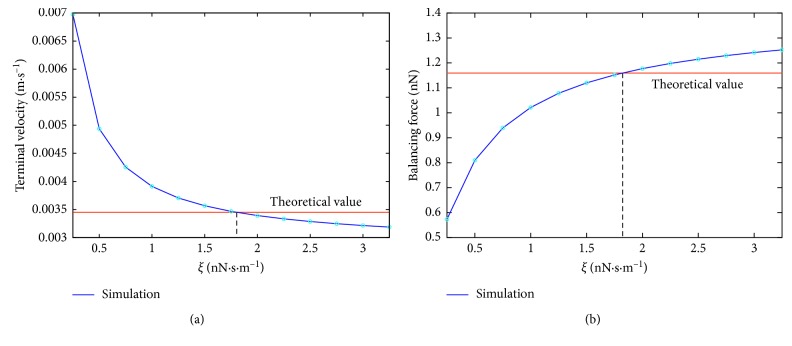
Influence of friction coefficient on (a) terminal velocity and (b) balancing force. Dotted lines indicate in both experiments the same correct value 1.82.

The simulation results are depicted in Figure 2. We can see how the increase in the friction coefficient changes the behaviour of the sphere in the simulations. In the case of terminal velocity experiment, increasing the friction causes decrease of the terminal velocity. This is natural, since increasing the friction coefficient means that the effect of fluid on the object is stronger and thus it slows the sphere down more for larger values of *ξ*.

In the case of the balancing force experiment, increasing the friction means again that fluid acts on the object stronger and thus we need larger balancing force to keep the object in place. The figures indicate that the expected values of terminal velocity and balancing force are achieved for the same value of friction coefficient, so

(11) $$\begin{array}{c} \xi_{\text{ref}}=1.82\;\mathrm{nN}\cdot s\cdot m^{-1}. \end{array}$$

## 5. Hypothesis for More General Shapes

In [31], we derived a renormalisation expression for computation of friction coefficient *ξ*_*n*,*r*_ for an arbitrary sphere with the number of mesh points *n* and radius *r*. The relation reads as

(12) $$\begin{array}{c} \xi_{n,r}=\frac{n_{\text{ref}}}{n}\frac{r}{r_{\text{ref}}}\xi_{\text{ref}}, \end{array}$$

where *ξ*_ref_ is the calibrated value for reference sphere with *n*_ref_ mesh points and radius *r*_ref_.

The question however remains what is the correct friction coefficient for nonspherical objects. The general function of the friction coefficient is to transfer the drag force of the fluid onto the object and back. Since the object is modelled by its membrane only, we need to transfer this drag force solely by the mesh points. Naturally, with more dense mesh, it is sufficient to transfer less force per mesh node to get the same effect on the membrane. It is thus logical to expect that friction coefficient inversely depends on *density* of the mesh points. This idea is supported by expression (12): increasing the number of mesh points while preserving the radius increases the *density* of mesh points and decreases the value of *ξ*.

Next, we need to define the *mesh density*. First approximation could be the number of mesh points per unit area, explicitly expressed by *n*/*S*, where *S* is the surface of the object. The relation (12) however suggests different: the definition of the *mesh density* is number of mesh points per unit length. In the case of spheres, the *mesh density* could be defined as *n*/*r*. For general shapes, we could choose the diameter of the object. This would, however, not reflect the fact that keeping the diameter and number of mesh points constant one can increase the surface, which subsequently decreases the density. Therefore, we suggest using square root of the surface and define *mesh density* as

(13) $$\begin{array}{c} \frac{n}{\sqrt{S}}. \end{array}$$

For spheres, for example, this choice is consistent (up to a constant) with radius. Our proposition is to use the following expression for computation of friction coefficient for nonspherical objects with *n* mesh points and surface *S*

(14) $$\begin{array}{c} \xi_{n,S}=\frac{n_{\text{ref}}}{n}\frac{\sqrt{S}}{\sqrt{S_{\text{ref}}}}\xi_{\text{ref}}. \end{array}$$

Note that for spheres, the newly proposed relation is consistent with (12). The formula is now shape independent.

## 6. Verification of Proposed Hypothesis for Ellipsoidal Shapes

**Figure 3:**
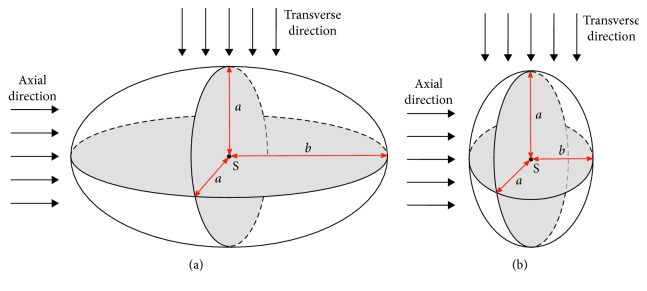
Ellipsoids with flow in axial and transverse directions. (a) Prolate ellipsoid with circular cross section with diameter *a* and prolonged radius *b*. (b) Oblate ellipsoid with circular cross section with diameter *a* and shortened radius *b*.

To verify the hypotheses, we use extended theoretical results concerning movement of rotationally symmetric ellipsoids in a fluid. Such ellipsoids are created from a sphere by prolonging (prolate ellipsoids) or shortening (oblate ellipsoids) of the sphere along one axis (Figure 3).

The relation (8) that is valid for spherical objects can be generalised for oblate and prolate ellipsoids [32]. The explicit expression for the drag force reads as

(15) $$\begin{array}{c} F_{\text{d}}=6\pi \nu aKv, \end{array}$$

where *ν* is the dynamic viscosity of the fluid, *v* is the relative velocity of the ellipsoid to the fluid, *a* is the radius of circular cross section of the ellipsoid, and *K* is the shape factor. *K* depends on the ratio *a*/*b* and on the flow direction. The concrete values of *K* for different cases are taken from [32] and shown in Table 1.

Using (15), we can reconstruct the theoretical steps from Sections 3.1 and 3.2 and conclude that *v*_*∞*_ for the terminal velocity experiment and *F*_A_ for the balancing force experiment read as

(16) $$\begin{array}{c} v_{\infty }=\frac{F_{0}}{6\pi \nu aK},\\ F_{\text{A}}=6\pi \nu aKv_{0}. \end{array}$$

These expressions give us expected values of *v*_*∞*_ and *F*_A_ in both experiments.

Now, we test our hypothesis. We choose 6 different ellipsoids (three of them are prolate and three are oblate). Each ellipsoid is triangulated using open source software GMSH [33]. The triangulation is regular, and thus, the local density of mesh points is approximately constant across the surface of the ellipsoid. The dimensions and other information are depicted in Table 2. Each of the six ellipsoids has different friction coefficient that is computed according to (14). The shape factor *K* was computed using expressions from Table 1.

Each ellipsoid is put in two different flows, one in an axial direction and one in a transversal direction. This results in 12 different scenarios. Each of these scenarios served as a starting point for both computational experiments, one for terminal velocity and one for balancing force. Altogether, we have thus computed 24 simulations. In these simulations, the size of the simulation box, elastic coefficients, and values for *F*_0_ and *v*_0_ were identical to those from the calibration of the reference sphere in Section 4.

Assuming that the hypothesis is correct, we should obtain the same values of expected *v*_*∞*_ and simulated *v*_*∞*_. Analogous statement holds for the expected balancing force *F*_A_ and simulated *F*_A_.

Using the corresponding friction coefficient, we have performed simulations for all 24 scenarios and we collected the computed values of terminal velocities and balancing forces, respectively. The actual results are presented in Table 3.

The Δ-columns contain relative differences. In the table, we can see that the simulated quantities (terminal velocity or balancing force) are fairly close to the expected values. The relative difference is always under 5%.

## 7. Dependence on Viscosity of the Fluid

Increasing viscosity means that with given velocity gradient, the shear stress increases. The friction coefficient is responsible for transfer of forces between the fluid and immersed objects, and thus, it is natural to expect dependence of the calibrated friction on viscosity. Our auxiliary simulations revealed that indeed the friction coefficient for the reference sphere is different for various viscosities. Therefore, we performed the sphere calibration from Section 4 for three typical fluids used in microfluidics. Natural choice is blood plasma. The values of blood plasma viscosity vary from 1.3 to 1.5  mPa · s [34, 35]. Other two fluids are phosphate-buffered saline suspensions used, for example, in [36, 37]. All three fluids have the same density 1025  kg · m^−3^, and their respective viscosities are presented in Table 4. The table shows the calibrated friction coefficient for the reference sphere with 393 nodes and radius 4  *μ*m. The relation (14) remains valid, with different values for the reference sphere.

## 8. Cell Flow in a Tube

To demonstrate the effect of different friction coefficients on real flow of cells, we performed a simulation of flow of a red blood cell exposed to Poiseuille flow in a tube with diameter comparable with the size of the cell. The parabolic profile of the fluid velocity means slower fluid velocities close to the tube wall and large velocity around the axis of the channel. The red blood cell when exposed to such flow deforms to a so-called parachute shape [3, 38, 39]. In this section, we show that our model with properly resolved fluid-structure interaction can capture this phenomenon.

We set up a simulation in a tube with diameter *r*=5  *μ*m and length 50  *μ*m. We consider fluid with density *ρ*=1000  kg · m^−3^ and viscosity 1.5  mPa · s flowing in a tube with volumetric flow rate 0.054  *μ*l · s^−1^. The elasticity of red blood cells is determined by its elastic coefficients. We take the following values:

(17) $$\begin{array}{c} k_{\text{s}}=0.006,\\ k_{\text{b}}=0.008,\\ k_{\text{al}}=0.001,\\ k_{\text{ag}}=0.5,\\ k_{\text{v}}=0.9, \end{array}$$

which correspond to the stretching experiments reported in [36]. Data from [36] have been used in numerous studies for validation of the computational models [3, 40, 41]. The friction coefficient computed from relation (14) was set to 1.56. This value was obtained for mesh with 374 nodes and RBC diameter 3.91.

**Figure 4:**
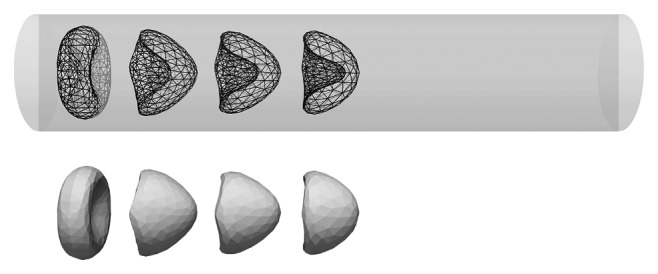
Parachute shape of the red blood cell in flow inside a tube. Snapshots of one cell at time instances 0ms, 1ms, 2ms, and 3ms.

Initial spatial orientation of the cell is transversal with respect to the axial direction. In Figure 4, snapshots of the shape are depicted in different time instances of the very same cell. In the figure, the cross section of the cell is visible. The cell gradually accelerates forming the parachute shape depicted in Figure 4. The shape resembles those reported in other computational and experimental studies [3, 38, 39].

When a different value of friction coefficient was used in the same experiment, the shape of the red blood cell was not affected. This seems to be in contradiction with results from previous sections where we claim that friction coefficient must be properly set depending on the mesh density and that we cannot choose its value arbitrarily. There is, however, a crucial difference between the terminal velocity and the balancing force experiments and the flow in an empty channel: in the empty channel, there is no external force exerted on the flowing object, and the object is being drifted by the fluid freely. There is thus much less transfer of force needed between the object and fluid compared with experiments, where external forces act against the movement of the object.

**Figure 5:**
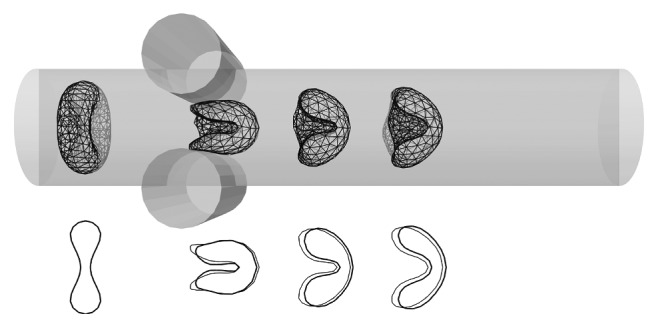
Constricted flow between two cylindrical obstacles. On top, the flow of cell with correct value of friction parameter 1.56 is depicted. Below, overlay of two simulations is shown: simulation with correct value of friction 1.56 (cell cross section with thin line) and simulation with lower value of friction 0.8 (cell cross section with thick line). Snapshots are depicted at time instances 0ms, 2ms, 3.5ms, and 5ms.

In situations, however, where the object does not flow freely in the flow, the friction coefficient must be set properly as demonstrated by the following example. We designed another test, where the cell is squeezed between two obstacles. Here, the obstacles substitute external forces by their influence on the cell. In Figure 5, two different simulations are depicted: one with the correct value of friction coefficient, 1.56 (cross section of the cell is drawn with thin line), and one with significantly lower value, 0.8 (cross section with thick line). We can clearly see different behaviour. After closer examination, one can identify two effects: different shape and delay.

Different shape is clearly visible at time instance 2  ms. With higher value of friction, the shape is more prolonged in axial direction than with lower value. This can be explained by larger transfer of force to the cell membrane. Near obstacles, the flow is almost zero due to no-slip condition, while in between the obstacles, the flow is fast. With larger friction, fluid near the obstacles decelerates the cell and in the middle it accelerates the membrane, causing more prolonged shape of the cell compared with the case with lower friction.

Delay is pronounced later, at 3.5ms and 5ms. This can be explained by the fact that when passing the obstacles, the no-slip condition causes more effective deceleration of the cell for higher friction, and thus for larger force transfer between static fluid and moving membrane.

## 9. Cell in a Shear Flow

Objects immersed in a shear flow exhibit complex behaviour. Movement of rigid ellipsoidal particles in a shear flow has been studied in [42]. The case of red blood cells is however different due to their irregularity and elasticity. Red blood cells exhibit several motion patterns in a shear flow. Under certain flow conditions, they may tumble or exhibit a tank-treading motion of the membrane or both, depending on the shear rate [4]. Above a certain threshold, the cell undergoes purely tank-treading motion. The membrane rotates around the cell's interior with a certain frequency. There are biological measurements of relation between the shear rate and the tank-treading frequency [43, 44].

Using these data, we will validate relation (14). We perform three sets of simulations for three different values of the friction coefficient. One value denoted by *ξ*_ok_ is the correct value computed from (14), while the other two values are defined as *ξ*_1_=0.5*ξ*_ok_ and *ξ*_2_=1.5*ξ*_ok_. The aim is to verify whether computations using *ξ*_ok_ give results corresponding to biological data, while computations using *ξ*_1_ or *ξ*_2_ give results diverging from the data.

**Figure 6:**
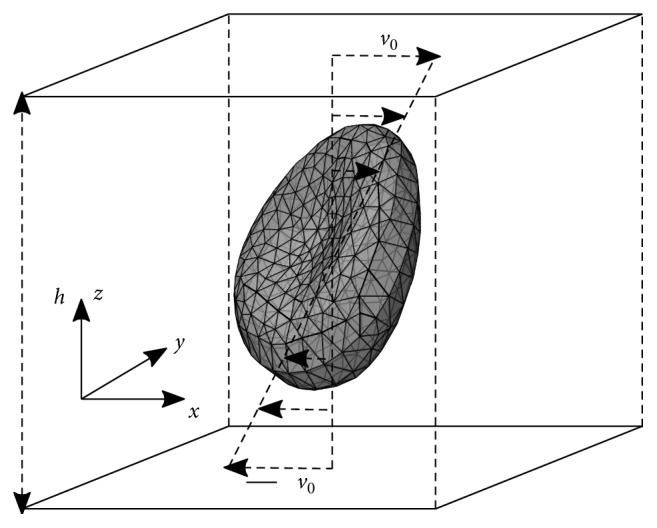
Two parallel walls move in opposite directions creating shear flow.

Shear flow can be induced between two parallel surfaces that move relative to one another. In practice, this means either one of them is stationary or the other moving or two plates moving with the same velocity in opposite directions, as depicted in Figure 6.

The shear rate $\dot{\gamma }$ generated by two walls moving with velocities *v*_0_ and −*v*_0_ can be computed from $\dot{\gamma }=2v_{0}/h$ with *h* being the distance between the walls. We will use a triangular mesh covering the surface of the cell with 393 mesh points and with surface area of approximately 142  *μ*m^2^. The model requires proper parameters so that the simulated cell has elasticity of a real red blood cell. The following values of the elastic parameters similar to those from [45] have been used in our computations:

(18) $$\begin{array}{c} k_{\text{s}}=6\;\;\mu \text{N/m},\\ k_{\text{b}}=3\times 10^{-19}\;\;\mathrm{Nm},\\ k_{\text{al}}=1\;\;\mu \text{N/m},\\ k_{\text{ag}}=0.5\;\;\mathrm{mN/m},\\ k_{v}=0.9\;\;{\text{mN/mm}}^{2}. \end{array}$$

We used a cubical computational domain with dimensions 20 × 20 × 20  *μ*m, *h*=20  *μ*m enclosing the cell located in its center. We use the following fluid properties to simulate suspending medium used in experiments from [43, 44]: *ρ*=1045  kg/m^3^, *ν*=10.7  mPa · s. The desired shear rate in the range from 0 to 200  s^−1^ can be generated by velocities ranging from 0 to 0.002  m/s. To obtain correct *ξ*_ref_, we extrapolate the values in Table 4 to get *ξ*_ref_=9.57 for *ν*=10.7  mPa · s. Finally, using (14), we can compute

(19) $$\begin{array}{c} \xi =\frac{n_{\text{ref}}}{n}\frac{\sqrt{S}}{\sqrt{S_{\text{ref}}}}\xi_{\text{ref}}=\frac{393}{393}\frac{\sqrt{142}}{\sqrt{201}}9.57=8.02. \end{array}$$

In all the experiments, we use time step of 0.1  *μ*m and the lattice grid of 2  *μ*m.

**Figure 7:**
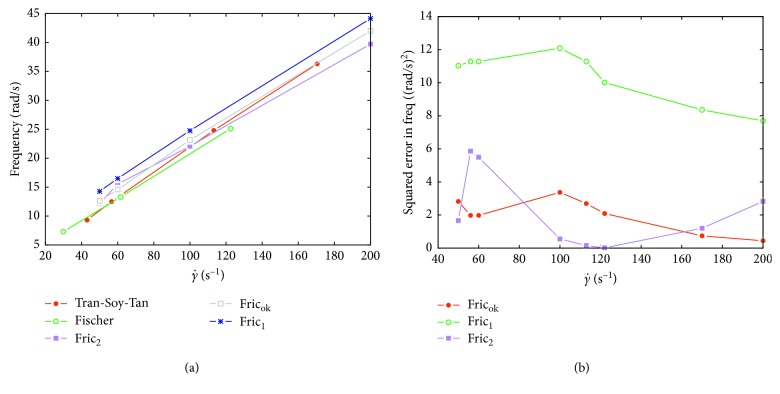
(a) Computed rotating frequencies versus experimental data. (b) Squared error of the computed data from the averaged biological data.

Computational results are summarized in Figure 7. In Figure 7(a), we can see that results for *ξ*_ok_ just slightly overshoot data from experiments. Clearly, the results for *ξ*_1_ overshoot the experimental data significantly. The results for *ξ*_2_ overshoot the experimental data in the range $\dot{\gamma }=50-100\;\;\mathrm{mPa}\cdot \text{s}$ while they undershoot the data in the range $\dot{\gamma }=100-200\;\;\mathrm{mPa}\cdot \text{s}$. To quantify the error, we plot squared error of simulated data from the averaged experimental data in Figure 7(b). Missing data points were either interpolated or extrapolated. The overall *L*_2_ error is 4.01 for *ξ*_ok_, 4.21 for *ξ*_2_, and 9.11 fro *ξ*_1_. This shows that the case for *ξ*_ok_ best fits the experimental data.

## 10. Conclusions

We analysed fluid-structure interaction that is based on a dissipative force between the fluid and structure. This interaction is mediated via friction coefficient between the mesh points and the fluid grid. In this work, we answered the question of proper value of the friction coefficient. The analysis was gradually established by first calibrating the reference sphere, generalisation to arbitrary sphere, proposal for arbitrary shape, validation of proposed hypothesis for ellipsoidal objects, and demonstration of validity for nonconcave objects, namely, for red blood cells.

Our study relies on explicit solutions to terminal velocity and balancing force experiments. Unfortunately, for asymmetrical or biconcave shapes, we are not aware of any such explicit solutions, and our study could not be extended to, for example, concave objects.

Nevertheless, we expect the relation (14) to be sufficiently accurate for general shapes. The reason for this is that the relation is based on local density of mesh points so that the force transfer between mesh points and surrounding fluid is locally the same over the whole surface of the object, regardless of its possible asymmetry and nonconvexity.

In case of red blood cells, their biconcave shape resembles ellipsoids quite well. The validity of the proposed results for red blood cells has been demonstrated in Section 8 by developing of typical parachute shape in a narrow tube. Furthermore, we have shown in simulations of red blood cell in a shear flow that the simulations with properly chosen friction coefficient correspond to real biological data of cell's rotating frequency. As soon as we set different friction coefficients, the computational data diverge from the biological measurements.

## Figures and Tables

**Table 1:** Values of the shape coefficient *K* appearing in the expression for the drag force.

Ellipsoid	*β*=	Flow	*K*
Prolate	*b*/*a*	Axial	$\frac{\left(4/3\right)\left(\beta^{2}-1\right)}{\left(\left(2\beta^{2}-1\right)/\sqrt{\beta^{2}-1}\right)\;\ln\left(\beta \;+\;\sqrt{\beta^{2}-1}\right)-\beta }$

Prolate	*b*/*a*	Transversal	$\frac{\left(8/3\right)\left(\beta^{2}-1\right)}{\left(\left(2\beta^{2}-3\right)/\beta^{2}-1\right)\;\ln\left(\beta \;+\;\sqrt{\beta^{2}-1}\right)\;+\;\beta }$

Oblate	*a*/*b*	Axial	$\frac{\left(4/3\right)\left(\beta^{2}-1\right)}{\left(\beta \left(\beta^{2}-1\right)/\sqrt{\beta^{2}-1}\right)\;\arctan\left(\sqrt{\beta^{2}-1}\right)\;+\;\beta }$

Oblate	*a*/*b*	Transversal	$\frac{\left(8/3\right)\left(\beta^{2}-1\right)}{\left(\beta \left(3\beta^{2}-2\right)/\sqrt{\beta^{2}-1}\right)\;\arctan\left(\sqrt{\beta^{2}-1}\right)-\beta }$

**Table 2:** The dimensions of 6 ellipsoids used to verify the hypothesis. The friction coefficient is recalculated by (14).

Ellipsoid	Type	Node	*a* (*μ*m)	*b* (*μ*m)	*S* (*μ*m^2^)	*ξ* (nN · s · m^−1^)
1	Oblate	594	3	1.5	78.05	0.75
2	Oblate	130	4	1	113.92	4.14
3	Oblate	1026	6	1.5	256.32	0.79
4	Prolate	130	5	8.75	479.50	8.50
5	Prolate	622	3	4.5	152.26	1.00
6	Prolate	986	6	10.5	690.48	1.34

**Table 3:** The simulation results of terminal velocity (given in m · s^−1^) and balancing force (given in nN) experiments for different ellipsoids put in axial and transverse flow.

Ellipsoid	Flow	*K*	Terminal velocity			Balancing force		
			Exp *v*_*∞*_	Sim *v*_*∞*_	Δ_*r*_*v*_*∞*_ (%)	Exp *F*_A_	Sim *F*_A_	Δ_*r*_*F*_A_ (%)
1	⟶	0.905	0.00508	0.00507	0.19	0.787	0.788	−0.14 %
2	⟶	0.867	0.00398	0.00407	−2.36	1.005	0.983	2.27
3	⟶	0.867	0.00265	0.00274	−3.36	1.508	1.456	3.45
4	⟶	1.153	0.00239	0.00235	1.77	1.671	1.691	−1.25
5	⟶	1.102	0.00418	0.00412	1.25	0.958	0.969	−1.17
6	⟶	1.153	0.00200	0.00191	4.04	2.005	2.066	−3.05
1	↓	0.793	0.00580	0.00562	3.20	0.689	0.712	−3.26
2	↓	0.682	0.00506	0.00490	3.02	0.791	0.817	−3.35
3	↓	0.682	0.00337	0.00335	0.69	1.187	1.195	−0.70
4	↓	1.288	0.00214	0.00214	0.27	1.866	1.858	0.43
5	↓	1.194	0.00385	0.00386	−0.20	1.038	1.035	0.28
6	↓	1.288	0.00179	0.00172	3.69	2.005	2.066	−3.05

**Table 4:** Calibrated friction coefficient for the reference sphere for three different fluids.

*ν*(mPa · s)	*ξ*(nN · s · m^−1^)
1.5375	1.82
1.3000	1.54
1.0000	1.18

## Data Availability

The findings of this study have been obtained using publicly available open source software ESPResSo, release 4.0, namely, its cell-flow module object-in-fluid [26]. The simulation data necessary to reproduce the results are included within this article. Further details are available from the corresponding author upon request.
